# Molecular detection and ultrastructure characterization of some drug-resistant Gram-negative bacteria isolated from bladder cancer patients

**DOI:** 10.1186/s12866-025-04572-6

**Published:** 2025-12-27

**Authors:** Mohamed M. A. Mousa, Mohamed I. Abou-Dobara, Mohamed M. El-Zahed, Manal E. El-Sherif, Hazem H. Saleh

**Affiliations:** 1 Department of Botany and Microbiology, Faculty of Science, Damietta University, New Damietta, 34517 Egypt; 2 Consultant of Medical Laboratory Analysis, Al-Shifa City Hospital, Gharbia, Egypt; 3 Urology and Nephrology Center, Mansoura University, Mansoura, Egypt

**Keywords:** Gram-negative bacteria, Genotypic, PCR, Ultrastructure, K. pneumoniae, E. coli, P. aeruginosa

## Abstract

**Background:**

Urinary tract infections (UTIs) are among the most common and potentially fatal illnesses that cancer patients experience. Infections in general are a major cause of morbidity and mortality in this population. The objective of this investigation was to detect some Gram-negative bacteria recovered from different urine specimens isolated from bladder cancer patients and examine their ultrastructural characteristics.

**Results:**

Gram-negative bacteria that are resistant to carbapenems were identified and isolated. Klebsiella pneumoniae, Escherichia coli, and Pseudomonas aeruginosa were then tested for employing meropenem in the phenotypic modified Hodge test (MHT), the modified carbapenem inactivation technique (mCIM), and the combined disk test (CDT). Genotypic detection of bacterial isolates was studied using *the blaNDM*,* blaVIM*, and *blaIMP* carbapenemase genes. 50 isolates (*K. pneumoniae*, *E. coli*, and *P. aeruginosa*) were tested for plasmid-mediated carbapenemase genes. Finally, agarose gel electrophoresis was used to investigate the polymerase chain reaction of *blaNDM*,* blaVIM*, and *blaIMP* carbapenemase genes. The structural characteristics of *E. coli*, *P*. *aeruginosa*, and *K. pneumoniae* were analyzed using transmission electron microscopy (TEM) to observe bacterial ultrastructure and illustrate cellular changes consistent with antimicrobial resistance. Gram-negative bacterial isolates included 35 *K. pneumoniae*, *E. coli*, and *P. aeruginosa* recovered from the study’s different urine samples. The resistance profile of *K. pneumoniae* and *E. coli* was greater than 80% for all tested antibiotics. Amikacin also exhibited high resistance at 80% (i.e., 20% susceptibility). The resistance profile of *P. aeruginosa* exceeded 100% for all examined antibiotics. Notably, amikacin was the single agent to which *K. pneumoniae* and *E. coli* demonstrated any measurable susceptibility (20%), distinguishing it as the best-performing antibiotic among the tested drugs, despite its overall limited efficacy. Unfortunately, high resistance was observed for all other examined antibiotics. The obtained results of MHT, mCIM, and CDT tests revealed that 82%, 32%, and 74% of isolated bacteria were carbapenem-resistant, respectively. PCR data revealed that *blaIMP* is the most relevant gene, followed by *blaNDM* and finally *blaVIM*, with 36%, 32%, and 22%, respectively. The current study showed a positive result of PCR for *blaNDM*,* blaVIM* genes, and *blaIMP*, respectively.

**Conclusions:**

Isolates that produced carbapenemase exhibited high levels of resistance to numerous antimicrobials employed in this investigation. The study focused on the genotypic detection of *blaNDM*,* blaVIM*, and *blaIMP*. To obtain a thorough understanding of the existence of high-risk clones with antimicrobial resistance, carbapenemase genes containing Gram-negative isolates were necessary. The study recommends that the evaluation of the importance of bacterial resistance in human therapies would undoubtedly reap major benefits upon the use of difficult-to-treat resistance (DTR) in clinical practice and at the bedside.

## Background

 Infections can disrupt treatment regimens, prolong hospitalization, increase healthcare costs, and reduce survival rates. Infections, especially those caused by bacteria, continue to be a leading cause of death, even if overall mortality rates have been falling. Knowledge of prevalent illnesses and patterns of antibiotic sensitivity is crucial for infection management, as is the use of appropriate empirical antimicrobial treatment [1]. Effective infection management is a significant challenge that necessitates a comprehensive understanding of the ever-evolving spectrum of infections. One of the most prevalent bacterial infections in humans is urinary tract infections (UTIs), which necessitate millions of visits to emergency departments, outpatient clinics, and hospitalizations each year. Sex, age, and specific predisposing factors all influence the prevalence of infection [2]. Urinary tract infections (UTIs) are inflammations brought on by harmful bacteria or viruses that infect the urinary tract. Factors that increase the risk of UTIs include age, sex, and lifestyle choices. Because the urethra is shorter and the bladder is more accessible in females, infections in this reproductive system are more common. Both community-acquired and hospital-acquired UTIs have different etiological agents [3]. Several studies have shown that inpatient and outpatient populations for UTI cases exhibit geographical diversity in the incidence of pathogens. Underlying tissue infections (UTIs) can be caused by a diverse range of enteropathogenic bacteria.

In community practice, *Escherichia coli* is the most common etiological agent. *Staphylococcus*,* Enterococcus*,* Proteus*,* Pseudomonas*,* Streptococcus*, and *Enterococcus faecalis* are among the other bacterial agents [4]. The majority of urinary tract infections (UTIs) in healthy, unrestricted urinary tracts are caused by enteric bacteria, especially *E. coli*. Various other types of bacteria, such as *S. saprophyticus*, *Enterococcus* spp., *Pseudomonas aeruginosa*, *Candida* spp., *Klebsiella pneumoniae*, *Proteus* spp., and *Enterobacter* spp., are also common in urinary tract infections [5]. The prevalence of UTIs caused by some bacteria is on the decline, according to previous research. On the other hand, yeasts, group *B streptococci*, and *K. pneumoniae* are on the rise. Furthermore, whereas UTIs caused by *Enterobacter* species have decreased, UTIs caused by *Acinetobacter* species and *P. aeruginosa* have increased [6].

Despite the importance of bacterial isolate identification in clinical microbiology laboratories, conventional phenotypic testing can be laborious and error-prone. Genotypic identification has now replaced more conventional approaches by making use of conserved sequences within genomic targets that provide information about evolutionary relationships. Standard bacterial culture is still the gold standard because these alternatives are more complicated, expensive, and require highly skilled workers [7]. So, this study’s goal was to identify certain Gram-negative bacteria that were isolated from bladder cancer patients and recovered from various urine specimens. Fifty of these isolates were Gram-negative bacteria. Among the Gram-negative bacteria isolated were five *Pseudomonas* species, ten *E. coli* species, and thirty-five *Klebsiella* species. In certain cases, a phase contrast microscope may be necessary to examine culture smears and germs. When a fresh bacterial strain should not have any stains, the phase is frequently the method of choice.

## Materials and methods

### Collection of clinical isolates

This study included 50 Gram-negative bacteria (GNB) isolated from the urine samples of patients with bladder cancer at UNC, Mansoura University, Egypt, from October 2021 to December 2022. Patients were instructed about the purpose and aim of the study and were allowed to obtain verbal and written informed consent, and patients had the right to refuse to participate in the study.

### Isolation of pathogenic bacteria from clinical samples

Bacterial colonies extracted from the culture plate were previously created using an inoculation loop and suspended in one milliliter of distilled water. The suspended bacterial solution was centrifuged for ten minutes at 5000 x g (7500 rpm). The supernatant was discarded, and the pellet was utilized for subsequent procedures. Each isolate was recognized through macroscopic and microscopic examination, and routine biochemical assays were conducted in accordance with Bergey’s Manual of Determinative Bacteriology [8].

### Determination of antimicrobial susceptibility test

Clinical and Laboratory Standards Institute (CLSI) recommendations were followed for the evaluation of antibiotic susceptibility in clinical isolates and the statistical analysis of carbapenemase producers (CPs). Antimicrobial compounds are serially two-fold diluted in matching media using the macrodilution method, which is also called the in-tube dilution test. The tubes are then supplemented with a known concentration of bacteria in suspension. Media turbidity allows visual assessment of MIC values after 24 h of incubation at 37 degrees Celsius, which measures bacterial growth.

Another macrodilution technique is the time-kill process. This test facilitates the assessment of the impact of varying concentrations of antimicrobial agents by analyzing the rate of bacterial mortality, thereby determining the bactericidal efficacy of these agents based on concentration and duration. Bacterial vitality is assessed by enumerating colonies on agar plates at consistent intervals over 24 h. The bacterial growth rate is assessed through variations in logCFU/mL during the initial 24-hour time-kill test. Experimental curves indicating the absence of growth or the lethal effect can be formed based on the results, providing insight into the interaction between the bacteria and the antimicrobial agent. The data might be undergoing additional analysis utilizing various mathematical models [9].

### Identification of pathogenic bacteria using the broth microdilution test

According to Hindler & Munro [10], the broth microdilution minimum inhibitory concentration (MIC) method is employed to semi-quantitatively assess the in vitro efficacy of an antimicrobial agent against bacterial isolates. The antimicrobial susceptibility test was conducted utilizing two varieties of VITEK cards: VITEK (bio-Merieux, Marcy l’Etoile, France) Card AST-N022 for antimicrobial susceptibility testing of non-lactose-fermenting, oxidase-positive Gram-negative bacilli, and AST-N020 for lactose-fermenting and non-lactose-fermenting, oxidase-negative Gram-negative bacilli. This test utilizes small quantities of broth delivered in sterile microdilution plates featuring conical bottom wells. Each well must contain 0.1 ml of broth. 0.1 (± 0.02) ml of antibiotic-containing broth was administered to each well. Incorporate a growth control well and a sterility control (uninoculated well). This work utilized commercially produced MIC test plates with dehydrated antimicrobial agents (dry-form plates) and validated them against a commercially prepared frozen-form plate, which serves as the CLSI/NCCLS reference method [11].

### Phenotypic detection of bacterial isolates

#### MHT

The procedure was carried out according to the instructions provided by CLSI (2015), which involved leaving the plates to dry for 15 min before placing ertapenem or meropenem discs in the middle of each plate. The tested isolates’ overnight cultures, which had 3–5 colonies, were streaked from the disc’s edge to the plates’ periphery after being incubated at 37 °C. Improved growth of the indicator strain of *E. coli*, which manifests as an indentation resembling a cloverleaf, was used to identify carbapenemase-producing isolates. To determine carbapenemase production among carbapenem-resistant isolates, MHT was performed in duplicate as an initial screening test [12].

#### mCIM

In 2019, the CLSI recommendations approved mCIM as a method for CP detection using commonly available laboratory reagents. Isolates of XDR GNB that could have been CPs were tested twice. The tested isolate was suspended in a meropenem disk and left to incubate for a minimum of four hours. We next moved the disk to a plate that had been inoculated with *E. coli* ATCC 25,922. Following an overnight incubation period, CPs were defined as tested isolates that displayed an inhibitory zone measuring 6–15 mm or colonies within 16–18 mm. In contrast, CPs were not defined as isolates with a zone of inhibition larger than 19 mm [8].

#### EDTA-enhanced carbapenem inactivation method (eCIM)

The eCIM was subsequently performed for all isolates to differentiate between serine-β-lactamase (SBL) and metallo-β-lactamase (MBL) production, as per CLSI guidelines. The eCIM procedure was identical to the mCIM, with the key exception that 20 µL of 0.5 M EDTA was added to the 2 mL Tryptic Soy Broth suspension before the meropenem disk was introduced. The results were interpreted by comparing them to the mCIM results:

- A positive mCIM result paired with a negative eCIM result (zone ≥ 19 mm) indicated the presence of an MBL.
- A positive result for both mCIM and eCIM indicated the presence of an SBL.

#### CDT

To provide a comprehensive phenotypic characterization of carbapenem resistance in our isolates, we evaluated three distinct assays. We compared the performance of the current CLSI-recommended Modified Carbapenem Inactivation Method (mCIM) with the historical Modified Hodge Test (MHT) and used an expanded Combined Disk Test (CDT) to screen for specific carbapenemase enzyme classes. An expanded Combined Disk Test (CDT) was performed to screen for the presence of Class A (KPC-type) and Class B (metallo-β-lactamase, MBL) carbapenemases. Following overnight culture, bacterial isolates were suspended to a turbidity equivalent to a 0.5 McFarland standard and swabbed to create a lawn on Mueller-Hinton agar plates.

Two different inhibitor combinations were tested:

1. For the detection of Class A carbapenemases, a 10-µg meropenem disk was placed 15–20 mm away from a meropenem disk supplemented with boronic acid.
2. For the detection of Class B (MBL) carbapenemases, a 10-µg imipenem disk was placed 15–20 mm away from an imipenem disk supplemented with EDTA.

After incubation at 37 °C for 18–24 h, a positive result was recorded if the inhibition zone diameter around the carbapenem-inhibitor disk was ≥ 5 mm larger than the zone around the carbapenem disk alone, as per published guidelines. Each test was performed in duplicate [6].

#### Gene detection by PCR

DNA extraction was done by utilizing the QIAamp^®^ DNA Mini Kit and Blood Mini Handbook, QIAGEN, GmbH, Germany. The thermal cycler (PerkinElmer model 9700) was programmed to perform PCR to detect antibiotic-resistant genes in different bacterial species [2].

#### Basic cycles for PCR amplification

PCR assay was run using the primers: blaIMP-1-F (5′ CTACCGCAGCAGAGTCTTTG − 3′) and blaIMP-1 R(5′ AACCAGTTTTGCCTTACCAT − 3′) and blaVIM primers: blaVIM F(TCTACATGACCGCGTCTGTC-3’) blaVIM R(5’-TGTGCTTTGACAACGTTCGC-3’) blaNDM primers: blaNDM F(5’-GGTTTGGCGATCTGGTTTTC − 3’) blaNDM R(5’- CGGAATGGCTCATCACGATC − 3’) as described by Kamel et al. [30] . The target DNA sequence is initially denatured at 95 °C for 5 min (1 cycle). Forty cycles of: A-denaturation (94 °C for 30 s), B-annealing (at 52 °C for 30 s) for (blaIMP-blaVIM-blaNDM) genes, C-extension (72 °C for 45 s), and Final extension (72 °C for 7 min) (1 cycle) (Kamel et al. 2018). The dye migrated to the final quarter of the gel, and the power was turned off after the current was activated at 80 volts for 2 h at 7.5 volts per centimeter of the gel. The gel was transferred to a UV transilluminator to ensure the integrity of the DNA. UV safety goggles were employed, and a photograph was captured using uncoated black and white Polaroid film in a Polaroid camera with a 4-second exposure duration. 0.8% agarose gel electrophoresis was employed to analyze the extracted DNA. PCR-mediated DNA amplification was conducted utilizing the extracted plasmids of phenotypically confirmed CPs as a template.

#### TEM

Two hours of fixation in glutaraldehyde buffered with 2.5% in 0.1 M PBS at a pH of 7.4 at 4 °C. PBS washing was performed three times, each time for ten minutes. After treatment with 1% osmic acid for thirty minutes. The process begins with washing three times with PBS for ten minutes each time, followed by dehydration with an escalating sequence of ethyl alcohol (30, 50, 70, 90%, and pure alcohol) for thirty minutes at each concentration, followed by infiltration with acetone for one hour. Araldite 502 resin was used to embed samples in the transmission electron microscope (TEM) after they had been dehydrated. Following the cutting of the plastic molds with the LEICA Ultra-cut (UCT ultra-microtome), the molds were stained with 1% toluidine blue. Following the inspection of semi-thin sections, ultra-thin sections were cut and stained with uranyl acetate. A JEOL-JEM-100 SX electron microscope from Japan, which is part of the Tanta University electron microscopy unit, was then used to inspect and photograph the samples after they had been counterstained with lead citrate.

### Statistical analysis

The statistical analysis was conducted using SPSS version 28.0 (IBM Corp., NY, USA), which was used for all statistical analyses and charts. The results were expressed as mean ± SE (mean ± standard error), the Chi-square test of independence, Fisher’s exact test for small sample sizes, and the Kappa statistic to measure the agreement between phenotypic and genotypic testing results. The value of *P* < 0.05 was used to indicate statistical significance.

## Results

### Identification of clinical isolates

During the study period, a total of 50 non-duplicate Gram-negative bacterial isolates were collected from various urine specimens. The collection comprised *K. pneumoniae* (*n* = 35), *E. coli* (*n* = 10), and *P. aeruginosa* (*n* = 5). The isolates were gathered using a convenience sampling method, whereby isolates identified during routine laboratory workflow on the days of collection were included in the study. All isolates were identified and corroborated using the hospital’s records.

### Antibiogram analysis of recovered GNB

Table 1 disclosed the resistance profiles of *K. pneumoniae*, *P. aeruginosa*, and *E. coli*. Exceeding 80% for piperacillin-tazobactam (TZP), cefotaxime (CTX), ceftazidime (CAZ), cefepime (FEP), imipenem (IMP), meropenem (MEM), ciprofloxacin (CIP), and nitrofurantoin (F). A significantly diminished resistance profile was seen for amikacin (AK) (80%). Table 1 indicates that the resistance profile of *Klebsiella* spp. was above 80% for TZP, CTX, CAZ, FEP, IMP, MEM, CIP, and F. A significantly lowered resistance profile for *Klebsiella* spp. and *E. coli* was observed for AK (80%) (Table 1). The results clearly demonstrate that all three bacteria are 100% resistant to antibiotics such as TZP, CTX, CAZ, FEP, IMP, and MEM, with some differences in resistance to AK and other antibiotics. The primary antimicrobial susceptibility of the bacterial and fungal isolates was determined using the VITEK-2 Compact system (bioMérieux, France). Susceptibility of the Gram-negative isolates was tested using GN (Gram-Negative) and AST-GN73 cards. Minimum inhibitory concentrations (MICs) were interpreted as Susceptible (S), Intermediate (I), or Resistant (R) according to the breakpoints defined by the Clinical and Laboratory Standards Institute (CLSI) document M100-S30, 2020. Daily quality control was performed using the appropriate reference strains [*E. coli* ATCC 25922] with all results falling within the acceptable ranges.

**Table 1:** Antimicrobial resistance patterns of MEM

Types of antibiotics	Percentage Resistance (%)					
	K. pneumoniae		E. coli		*P*. aeruginosa	
	Resistance %	Sensitive%	Resistance %	Sensitive%	Resistance %	Sensitive%
TZP	100%	0%	100%	0%	100%	0%
CTX	100%	0%	100%	0%	100%	0%
CAZ	100%	0%	100%	0%	100%	0%
CEF	100%	0%	100%	0%	100%	0%
IMP	100%	0%	100%	0%	100%	0%
MEM	100%	0%	100%	0%	100%	0%
AK	80%	20%	80%	20%	100%	0%
CIP	100%	0%	100%	0%	100%	0%
F	100%	0%	100%	0%	100%	0%

### Phenotypic detection of bacterial isolates

**Fig. 1:**
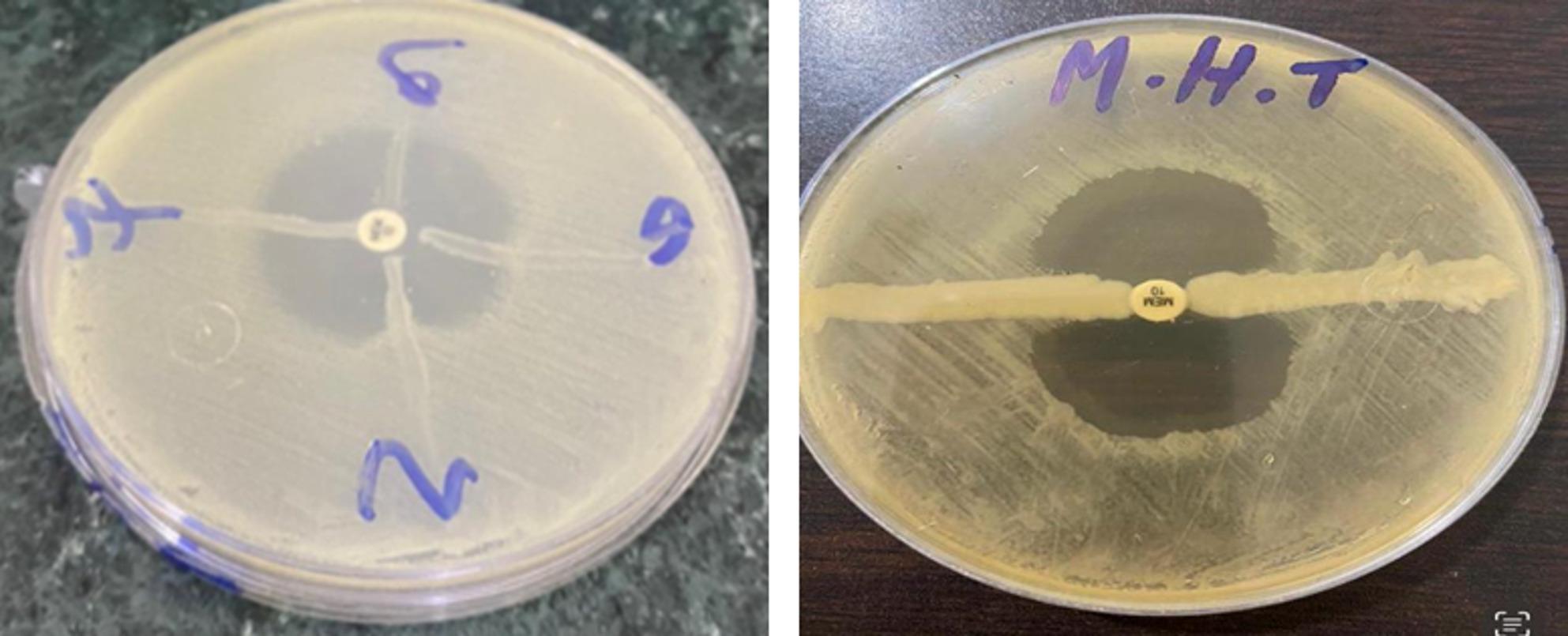
Phenotypic detection of bacterial isolates using MHT

*Pseudomonas aeruginosa*, *K. pneumoniae*, and *E. coli* were among the Gram-negative bacterial isolates tested genetically. We used MHT, mCIM, and CDT to find out whether *K. pneumoniae*, *E. coli*, and *P. aeruginosa* could make carbapenemases. Screening for the prevalence of Gram-negative bacterial isolates resistant to MHT, mCIM, and CDT (*K. pneumoniae*, *E. coli*, and *P. aeruginosa*). We used MEM to do phenotypic detection on bacterial isolates. Improved *E. coli* growth, visible as a cloverleaf indentation, was indicative of carbapenemase-producing isolates, whereas the lack of *E. coli* growth along the streak of tested isolates was indicative of non-carbapenemase-producing isolates. Carbapenemase production among carbapenem-resistant isolates was initially determined by doing Fig. 1 in duplicate. Isolates that yielded negative results were retested to seek confirmation.

**Fig. 2:**
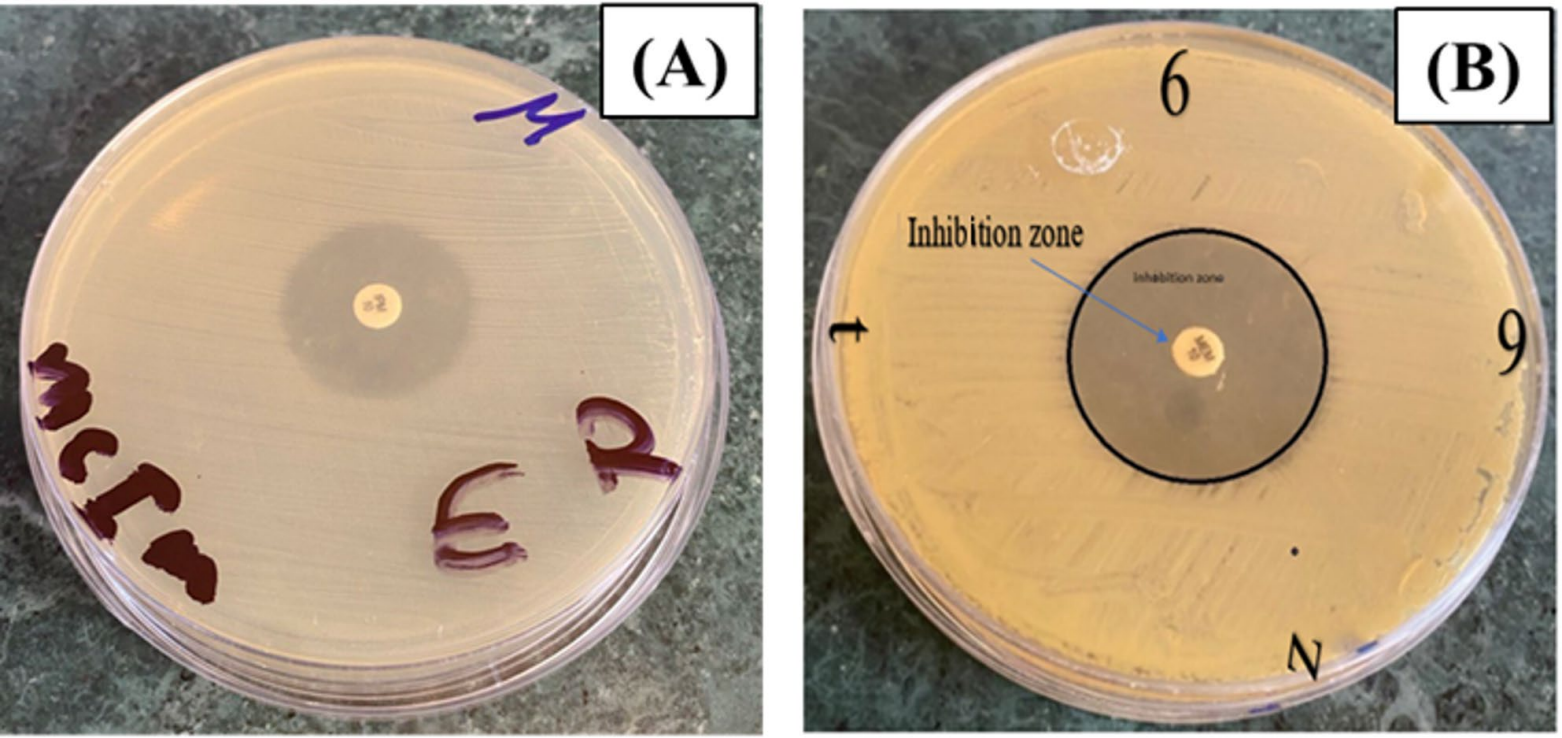
Phenotypic detection of bacterial isolates using the mCIM test. **A** Positive result (11 mm). **B** Positive result (20 mm). Positive mCIM test showed inhibition zone after incubation

The mCIM test revealed inhibition zones ranging from 6 to 15 mm, which suggests a positive result, indicating carbapenemase production by the test organisms (Fig. 2).

**Fig. 3:**
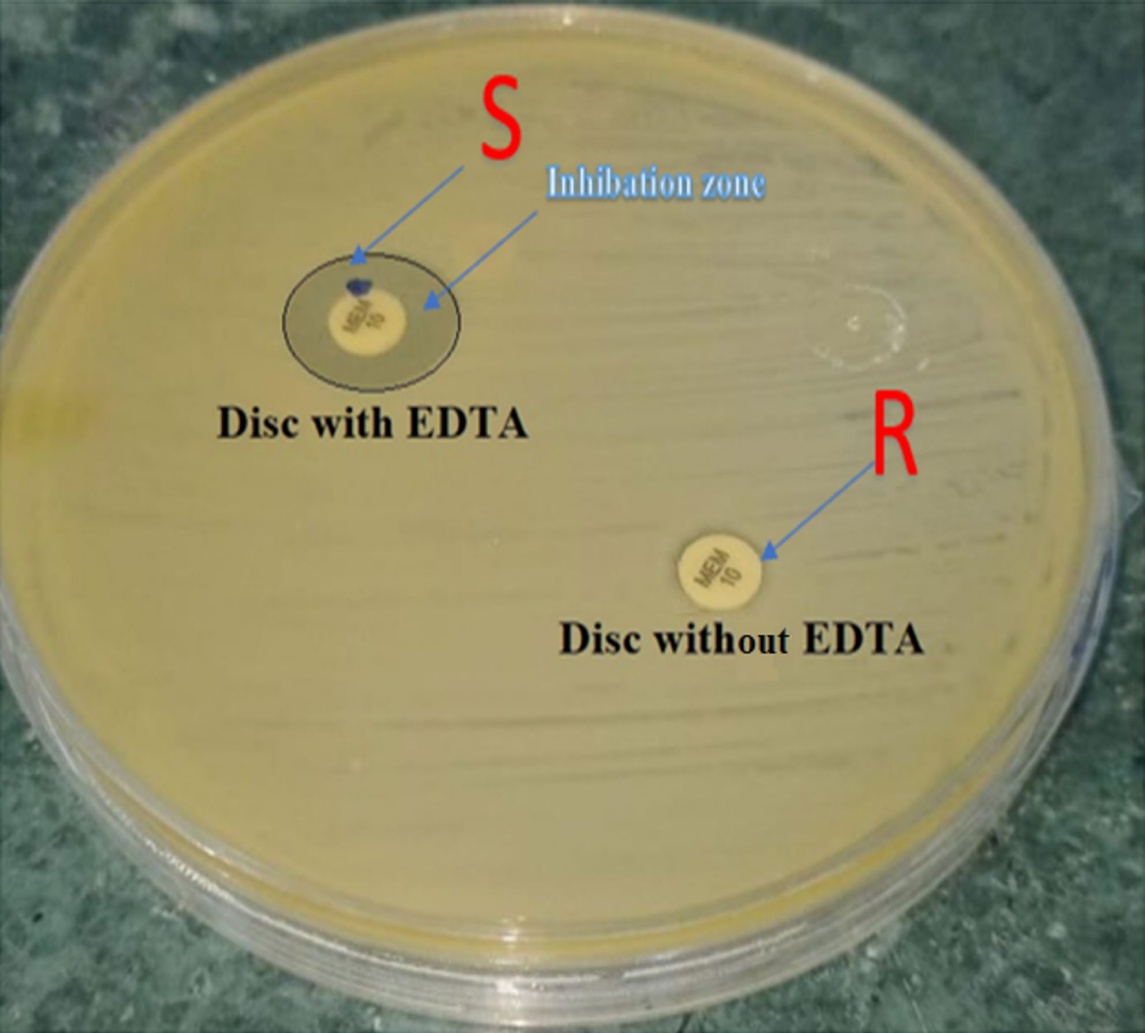
Phenotypic detection of bacterial isolates using CDT. Combined disk diffusion test (CDT), Positive results showed as an imipenem disc without EDTA, no inhibition zone, and an imipenem disc with EDTA producing an inhibition zone

A confirmatory test for carbapenemase production was declared positive when the CDT increase of the inhibition zone (≥ 5 mm) of the carbapenem disk with inhibitor was greater than that of carbapenem alone during overnight incubation (Fig. 3). The results showed an inhibition zone diameter ≥ 5 mm, indicating resistance against the MEM disc, while sensitive results occurred against the MEM disc with EDTA.

The results of the MHT test revealed that 82% of the examined Gram-negative bacteria isolates were carbapenem-resistant (Tables 2 and 3). According to the mCIM test, our results demonstrated that 32% of the examined Gram-negative bacteria isolates were carbapenem-resistant. According to the CDT test, our results demonstrated that 74% of the examined Gram-negative bacteria isolates were carbapenem-resistant.

**Table 2:** Phenotypic detection pattern of bacterial isolates

Phenotypic Test	Number of Positive Isolates (*n* = 50)	Percentage (%)
Modified Hodge test (MHT)	41	82%
Modified carbapenem inactivation method (mCIM)	16	32%
Combined disk test (CDT)	37	74%

**Table 3:** Phenotypic carbapenem resistance detection

Phenotypic carbapenem resistance detection	MHT	mCIM	CDT	*P* value
	80%	30%	77%	< 0.001

As shown in Table 4, genotypic detection of bacterial isolates using *blaNDM*,* blaVIM*, and *blaIMP* carbapenemase genes. Out of 50 isolates, 10 *E. coli*, 35 *Klebsiella* sp., and 5 *Pseudomonas* sp. were tested for plasmid-mediated carbapenemase genes. The result of PCR reveals that *blaNDM* showed 32% carbapenem resistance, where 16 isolates were positive of the total 50 samples; *blaIMP* showed 36% carbapenem resistance, where 18 isolates were positive of the total 50 samples; and *blaVIM* showed 22% carbapenem resistance, where 11 isolates were positive of the total 50 samples.

**Table 4:** Genotypic carbapenem detection of bacterial isolates

Genotypic detection of bacterial isolates	Organism	No of isolates	Percentage of carbapenem resistance (%)	*P* value
*blaNDM*	***K. pneumoniae (35)***	14	32% (16/50)	0.12
	***E. coli (10)***	1		
	***P. aeruginosa (5)***	1		
*blaIMP*	***K. pneumoniae (35)***	15	36% (18/50)	
	***E. coli (10)***	1		
	***P. aeruginosa (5)***	2		
*blaVIM*	***K. pneumoniae (35)***	10	22% (11/50)	
	***E. coli (10)***	0		
	***P. aeruginosa (5)***	1		

### Molecular characterization

**Fig. 4:**
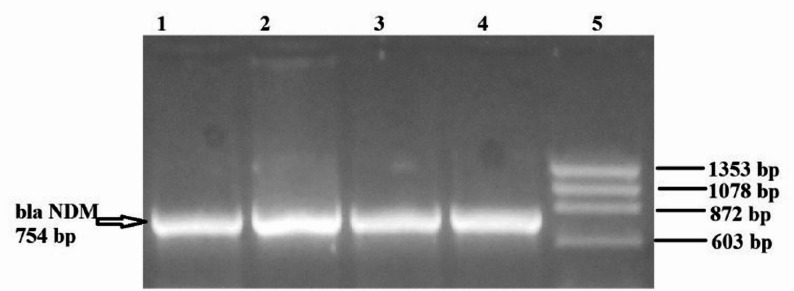
Agarose gel electrophoresis showing results of genotypic detection of bacterial isolates using *blaNDM* carbapenemase genes. Lanes 1, 2, 3, and 4 are positive for *blaNDM* at 754 bp, and lane 5 DNA ladder (ΦX174)

Figure 4 demonstrates genotypic detection of bacterial isolates using *blaNDM* DNA extraction via PCR and visualized using gel electrophoresis, in which lanes 1, 2, 3, and 4 are positive for *blaNDM* at 754 bp, and lane 5 is the DNA ladder (ΦX174).

**Fig. 5:**
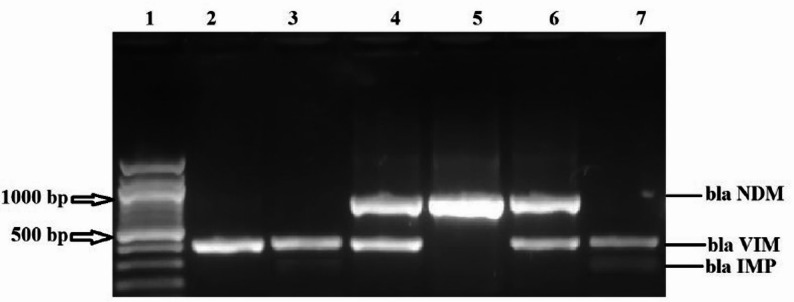
Agarose gel electrophoresis showing results of genotypic detection of bacterial isolates using *blaNDM*,* blaVIM*, and *blaIMP* carbapenemase genes (multiplex for 3 genes). Lane 1 is a DNA ladder (ΦX174). Lanes 2 and 3 are positive for blaVIM. Lanes 4 and 6 are positive for both VIM and NDM. Lane 5 is positive for blaNDM. Lane 7 positive for blaVIM

Figure 5 demonstrates genotypic detection of bacterial isolates using *blaNDM*, *blaVIM*, and *blaIMP* carbapenemase genes, DNA extraction via PCR, and visualization using gel electrophoresis, in which lane 1 is a DNA ladder (ΦX174), while lanes 2 and 3 are positive for *blaVIM*. Lanes 4 and 6 are positive to both VIM and NDM, lane 5 is positive to *blaNDM*, and lane 7 is positive to *blaVIM*.

Tables 5 and 6 demonstrate the relationship between phenotypic characteristics and their genotypic tests

**Table 5:** Summarization of phenotypic and genotypic characters of Gram-negative isolates

MHT	mCIM	CDT	blaNDM	blaIMP	bla VIM	NO. of isolate		
						K. pneumoniae	E. coli	*P*. aeruginosa
+ve	+ve	+ve	+ve	+ve	+ve	**2**	**-**	**-**
-ve	-ve	-ve	-ve	+ve	-ve	**1**	**-**	**-**
+ve	-ve	-ve	-ve	+ve	-ve	**-**	**1**	**-**
-ve	-ve	+ve	-ve	-ve	-ve	**2**	**-**	**-**
-ve	-ve	-ve	+ve	+ve	+ve	**1**	**-**	**-**
+ve	-ve	+ve	+ve	+ve	+ve	**1**	**-**	**-**
+ve	-ve	+ve	-ve	+ve	-ve	**3**	**-**	**1**
+ve	-ve	+ve	+ve	+ve	-ve	**2**	**-**	**-**
+ve	-ve	-ve	-ve	-ve	-ve	**2**	**1**	**-**
+ve	-ve	+ve	+ve	-ve	+ve	**1**	**-**	**-**
+ve	+ve	-ve	+ve	+ve	+ve	**1**	**-**	**-**
+ve	-ve	+ve	+ve	-ve	-ve	**2**	**-**	**1**
+ve	-ve	+ve	-ve	-ve	-ve	**1**	**7**	**-**
+ve	-ve	+ve	-ve	+ve	+ve	**3**	**-**	**-**
-ve	+ve	-ve	-ve	-ve	-ve	**1**	**-**	**-**
+ve	+ve	+ve	-ve	-ve	-ve	**4**	**-**	**-**
+ve	+ve	+ve	+ve	-ve	-ve	**4**	**1**	**-**
-ve	-ve	-ve	-ve	-ve	-ve	**-**	**-**	**2**
-ve	-ve	+ve	-ve	+ve	+ve	**1**	**-**	**1**
+ve	+ve	-ve	-ve	-ve	-ve	**3**	**-**	**-**

**Table 6:** Summary of phenotypic and genotypic resistance in Gram-negative isolates

	Total isolate	Phenotypic resistance	Genotypic resistance
*K. pneumoniae*	35	16	10
*E. coli*	10	7	3
*P. aeruginosa*	5	0	2

The comparison between MHT and mCIM reveals slight agreement (Kappa = 0.19, 95% CI: 0.03; 0.34), with a *p*-value of 0.048 (Table 7). The MHT vs. CDT comparison demonstrates substantial agreement (Kappa = 0.77, 95% CI: 0.64; 0.89) with a *p*-value of 0.03. Lastly, the mCIM vs. CDT comparison shows fair agreement (Kappa = 0.28, 95% CI: 0.05; 0.51) with a *p*-value of 0.005. 

**Table 7:** Cohen’s kappa value

Test Comparison	Cohen’s Kappa (95% CI)	Agreement	*p*-value
MHT vs. mCIM	0.19 (0.03; 0.34)	Slight	0.048
MHT vs. CDT	0.77 (0.64; 0.89)	Substantial	0.03
mCIM vs. CDT	0.28 (0.05; 0.51)	Fair	0.005

### TEM analysis of *E. coli* during the mCIM test

**Fig. 6:**
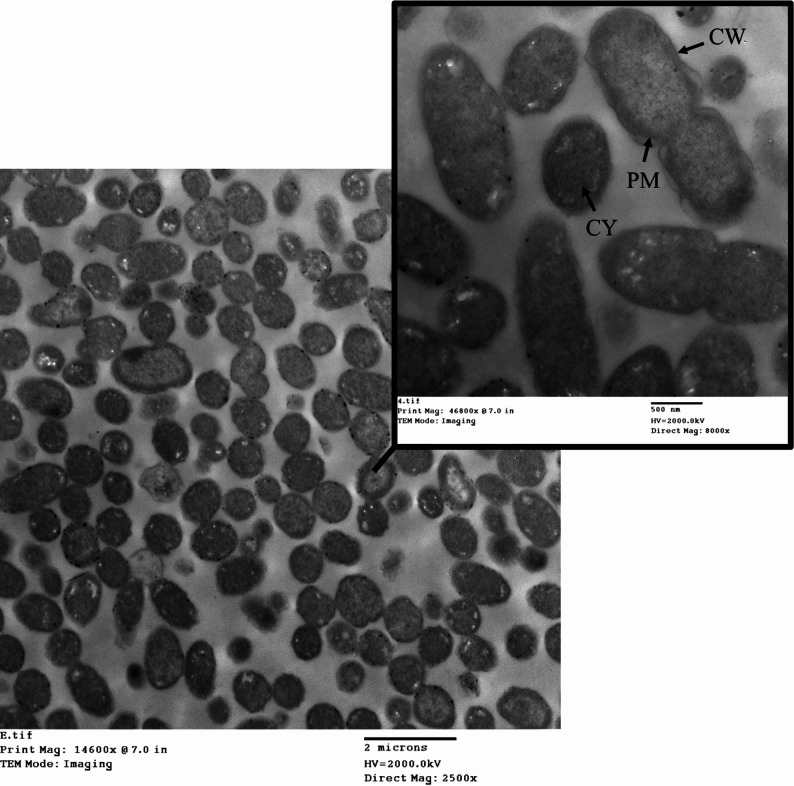
TEM imaging of *E. coli* before the mCIM test. CW is the cell wall. CM is the cytoplasmic membrane. CY is cytoplasm

TEM imaging of *E. coli* before the mCIM test showed intact cell walls and membranes, with a uniform rod-like shape and well-preserved internal structures (Fig. 6). A typical Gram-negative appearance with a relatively thin peptidoglycan layer and outer membrane, periplasmic space, and flagella. No significant distortions, intact membrane, and visible cell wall.

**Fig. 7:**
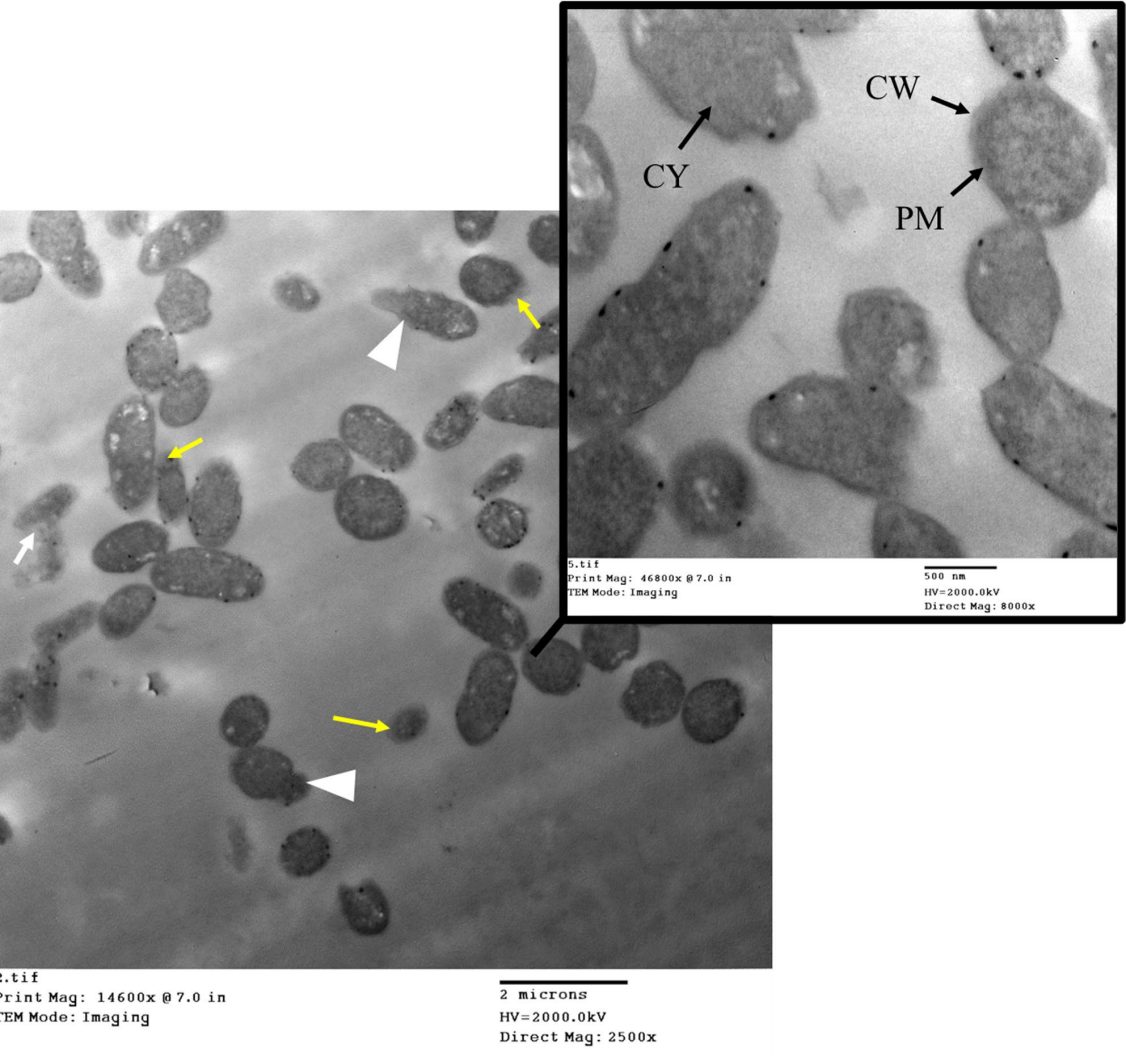
Transmission electron microscopy (TEM) of carbapenemase-producing *E. coli* after meropenem exposure. The image illustrates ultrastructural changes consistent with antibiotic-induced stress, including significant loss of the typical rod shape (white arrowhead), evidence of membrane damage (white arrows), and cytoplasmic material leakage (yellow arrows) in the *E. coli* isolate. CW is the cell wall. CM is the cytoplasmic membrane. CY is cytoplasm

To investigate the morphological effects of meropenem, a carbapenemase-producing *E. coli* isolate was exposed to the antibiotic and examined using TEM. The analysis revealed that significant cellular damage still occurs while the enzyme likely inactivates some of the antibiotic, allowing for survival. The cell wall often showed signs of damage or thinning. As seen in Fig. 7, the *E. coli* cells exhibited severe membrane damage and rupture. In many cells, the cytoplasm appeared shrunken, and internal structures like ribosomes were disorganized. In the most severe cases, cells showed signs of collapse and lysis due to the breakdown of the cell wall and membrane integrity.

### TEM analysis of *K. pneumoniae* during the mCIM test

**Fig. 8:**
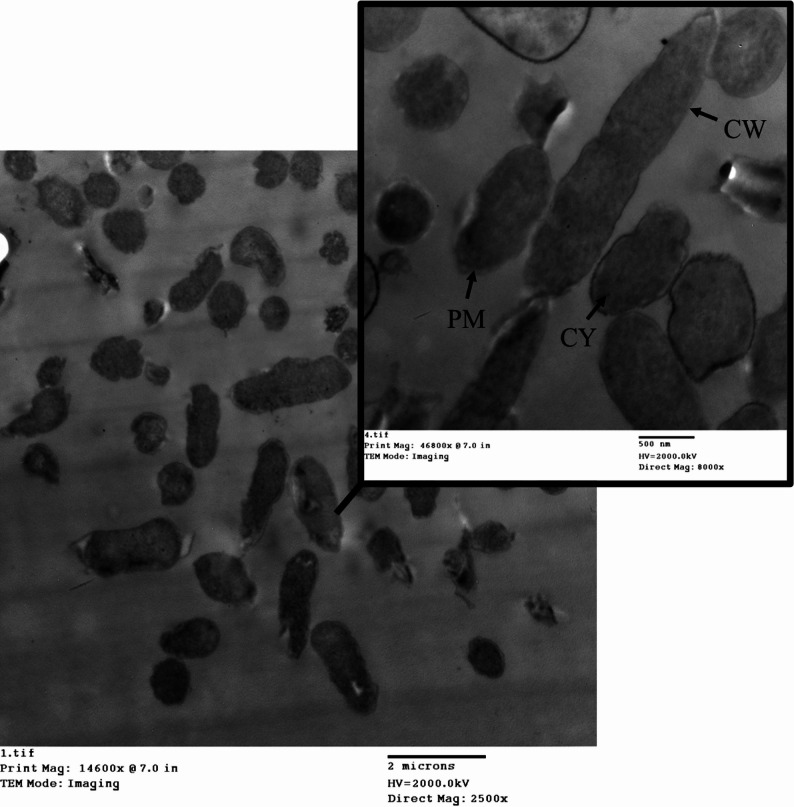
TEM imaging of *K. pneumoniae* before the mCIM test. CW is the cell wall. CM is the cytoplasmic membrane. CY is cytoplasm

TEM imaging of *K. pneumoniae* before the mCIM test showed a well-preserved cell wall and membrane, with no visible structural damage. Gram-negative with a thicker cell wall, a prominent capsule (often visible around the cell), and an outer membrane. The capsule is thick and could obscure the cell shape (Fig. 8).

**Fig. 9:**
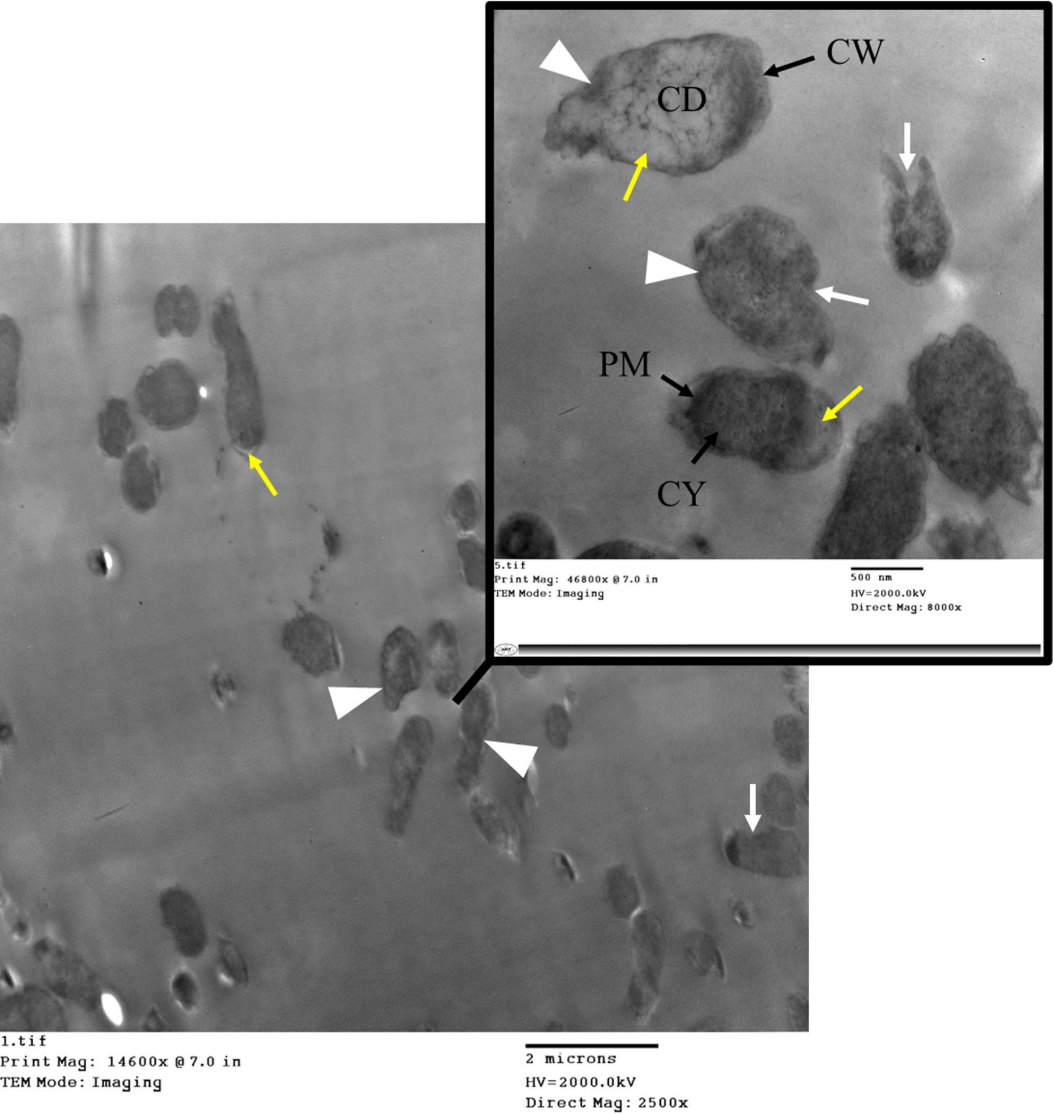
TEM imaging of carbapenemase-producing *K. pneumoniae* after exposure to MEM during mCIM. The image illustrates significant ultrastructural changes in the bacterial cells, which are consistent with severe cellular stress and possible death. Key observations include highly condensed and irregular cytoplasmic material, significant loss of the typical rod shape (pleomorphism), and widespread cellular disintegration (lysis). These morphological findings are observational and correlate with the bacteria’s inability to survive the combination of carbapenemase production and meropenem exposure during the mCIM test. CW is the cell wall. CM is the cytoplasmic membrane. CY is cytoplasm

Ultrastructural analysis of the carbapenemase-producing *K. pneumoniae* isolate subjected to meropenem (MEM) during the mCIM procedure revealed profound morphological changes (Fig. 9). After the mCIM test, the *K. pneumoniae* cell wall displayed structural degradation or thinning, and the capsule could be disrupted. Increased membrane damage was also observed. The overall cell shape became more irregular or deformed. The TEM images showed bacteria with highly irregular shapes (pleomorphism), significant condensation of the cytoplasmic contents, and distinct evidence of cellular disintegration (lysis). These observational findings illustrate the severe impact of meropenem on the bacterial integrity, consistent with cellular stress and death, rather than demonstrating a direct, causal mechanism of resistance.

### TEM analysis of *P. aeruginosa* during the mCIM test

**Fig. 10:**
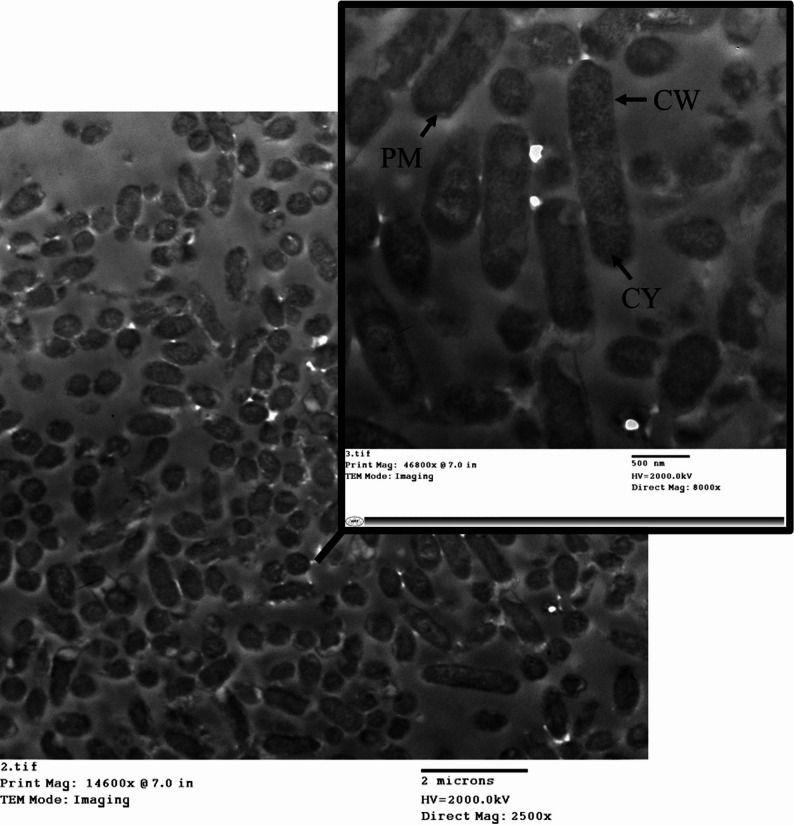
TEM imaging of *P. aeruginosa* before the mCIM test. CW is the cell wall. CM is the cytoplasmic membrane. CY is cytoplasm

TEM imaging of *P. aeruginosa* before the mCIM test showed a well-preserved cell wall and membrane. Typical Gram-negative structure with a thinner peptidoglycan layer, outer membrane with lipopolysaccharides, and flagella. Cell wall intact, flagella visible, and no significant morphological distortions (Fig. 10).

**Fig. 11:**
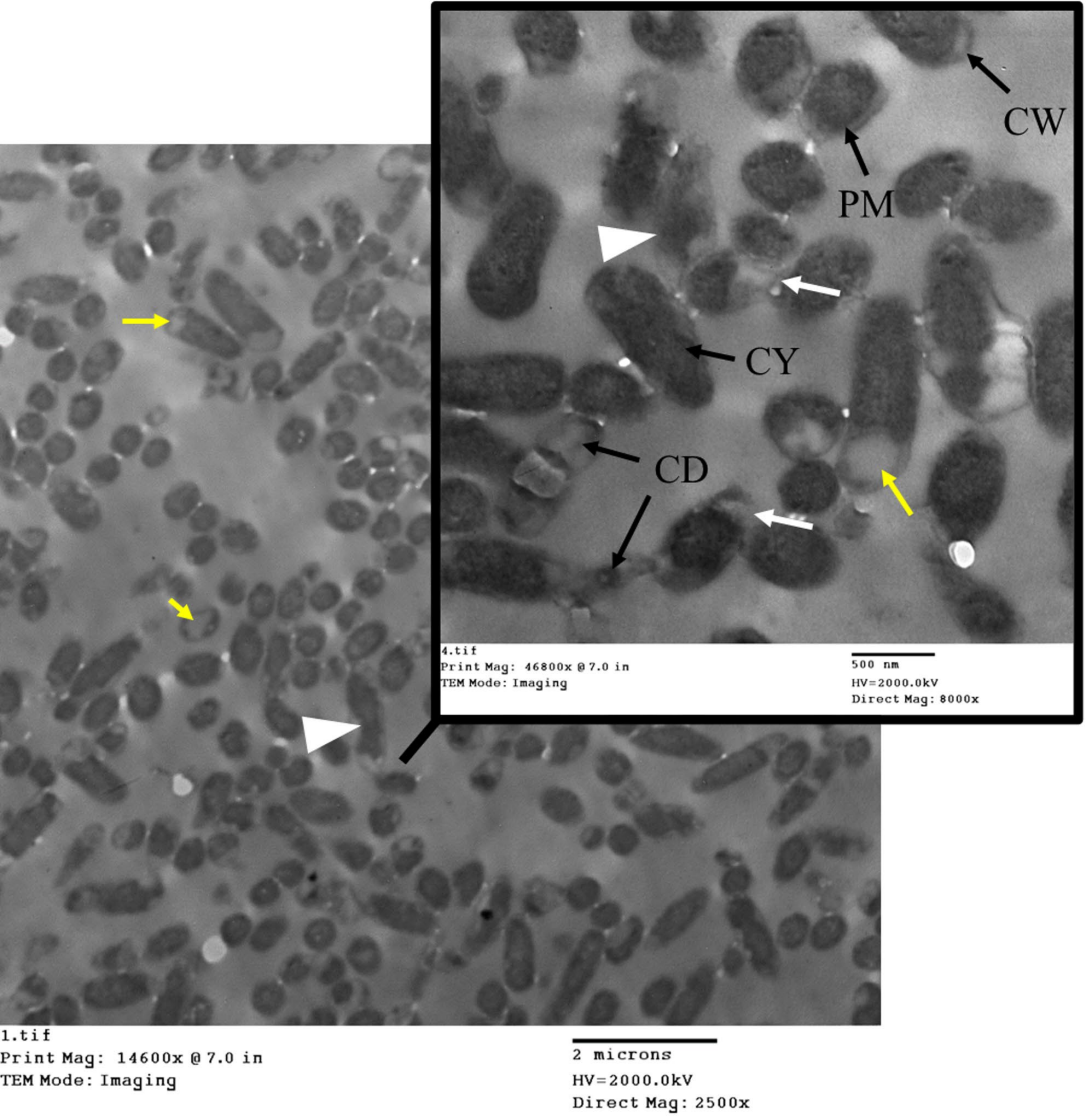
TEM imaging of carbapenemase-producing *P. aeruginosa* after exposure to MEM during mCIM. The images reveal ultrastructural changes that correlate with high cellular stress following antibiotic exposure. Yellow arrows indicate cytoplasmic material leakage and dehydration. White arrowheads show areas of irregular cell shapes (pleomorphism), where the characteristic morphology is lost. White arrows highlight regions of membrane damage, which may compromise cellular integrity. CD encloses severely damaged cells. CW is the cell wall. CM is the cytoplasmic membrane. CY is cytoplasm

Ultrastructural analysis of the *P. aeruginosa* isolate after meropenem exposure was performed using TEM (Fig. 11). *P. aeruginosa* displayed distorted or ruptured cell membranes, showing signs of cytoplasm leakage. The cell shape appeared irregular (pleomorphism), and signs of flagella degradation or detachment were noted. The outer membrane could also show signs of thinning or disruption. Furthermore, the structural analysis showed instances of membrane blebbing or damage and the presence of smaller, lightly dense cells, which are morphologically consistent with severely damaged or senescent cells.

### TEM analysis of *P. aeruginosa* during CDT testing

**Fig. 12:**
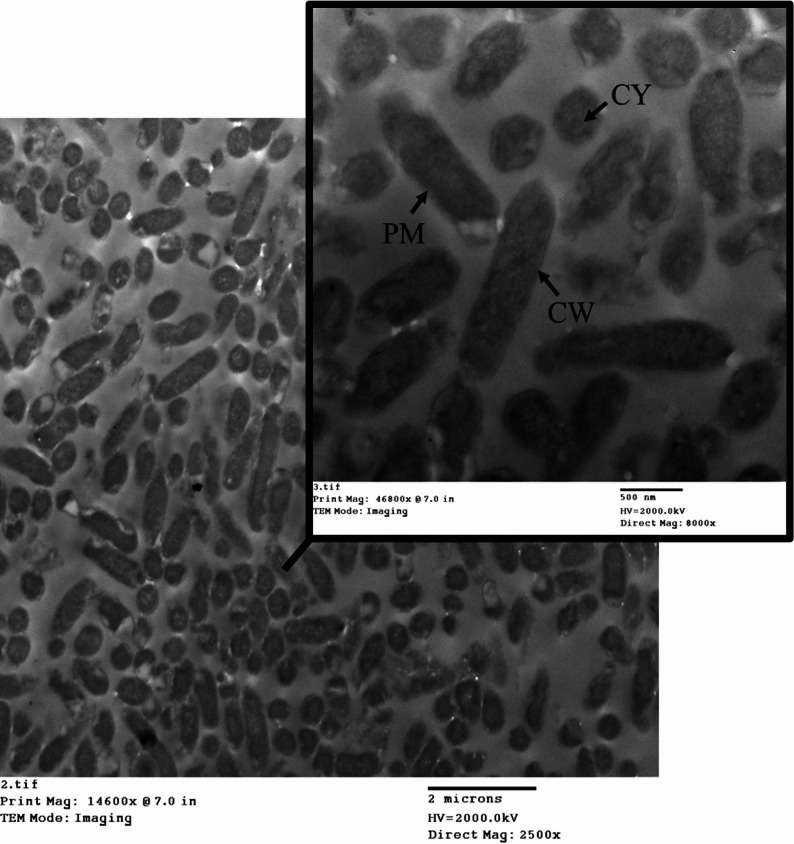
TEM imaging of *P. aeruginosa* before CDT. CW is the cell wall. CM is the cytoplasmic membrane. CY is cytoplasm

TEM analysis of *P. aeruginosa* before CDT reveals a typical Gram-negative morphology with a straight rod shape. The outer membrane has a regular bilayer structure, and the cytoplasm is dense (Fig. 12).

**Fig. 13:**
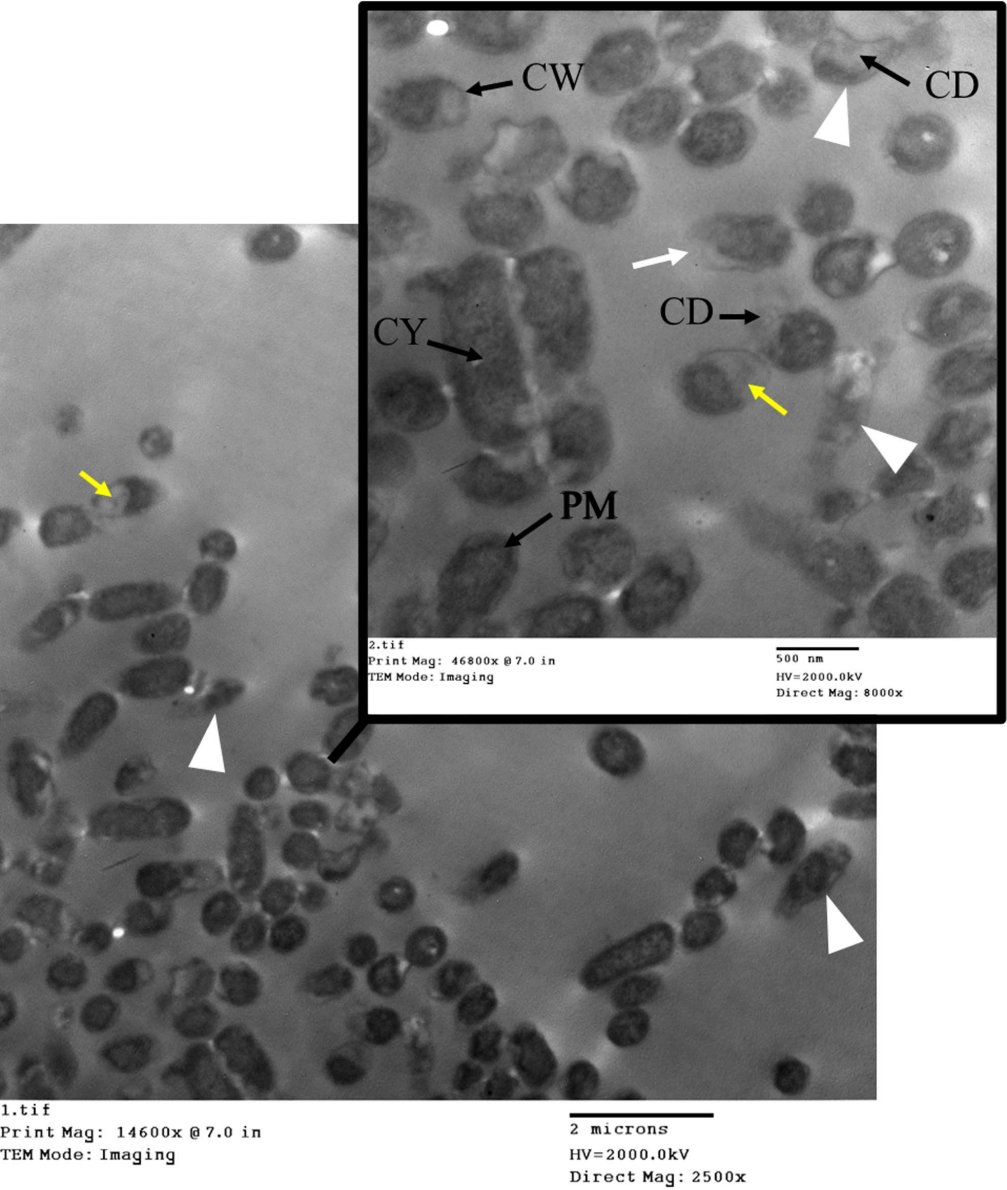
TEM imaging of *P. aeruginosa* after exposure to CDT. The images reveal ultrastructural changes that correlate with severe cellular stress following exposure to the combined antibiotic agents. Yellow arrows point to significant cellular dehydration and distress. White arrowheads indicate areas of irregular cell shapes (pleomorphism), where the typical rod morphology is severely distorted. White arrows highlight regions showing membrane disruption. CW is the cell wall. CM is the cytoplasmic membrane. CY is cytoplasm

Ultrastructural analysis of the *P. aeruginosa* isolate after exposure to the CDT conditions was performed using TEM (Fig. 13). Following exposure to CDT, *P. aeruginosa* shows significant structural alterations. Observed features included pleomorphism, resulting in severely irregular cell shapes, and noticeable lysis of the cytoplasmic material. The outer membrane begins to exhibit blebbing and distortion, likely due to damage caused by antibiotic exposure. The cytoplasm appears less organized, indicating stress or damage. Additionally, the periplasmic space was significantly expanded due to the breakdown of the bacterial cell wall and loss of cellular contents. However, the population response appeared heterogeneous, with some cells retaining a more intact morphology. These observational findings are consistent with the known cytotoxic effects of antibiotics on Gram-negative bacteria and correlate with the overall susceptibility/resistance profile observed.

### TEM analysis of *K. pneumoniae* during CDT testing

**Fig. 14:**
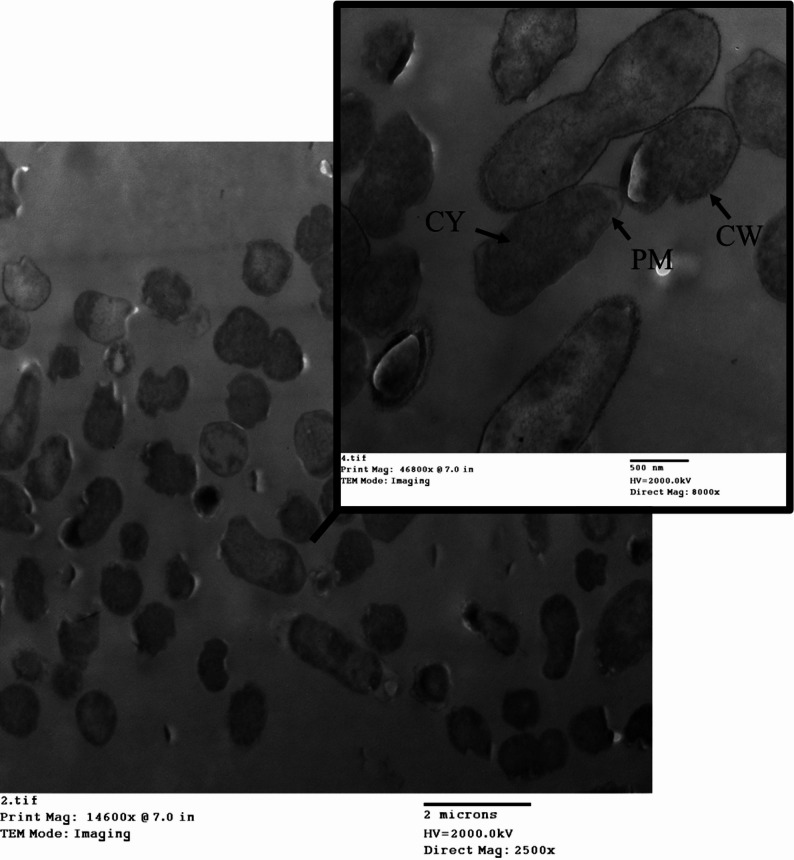
TEM imaging of *K. pneumoniae* before CDT. CW is the cell wall. CM is the cytoplasmic membrane. CY is cytoplasm

TEM analysis of *K. pneumoniae* reveals its characteristic encapsulated, Gram-negative rod morphology. The outer membrane appears intact with a regular bilayer structure, and the cytoplasm is dense (Fig. 14).

**Fig. 15:**
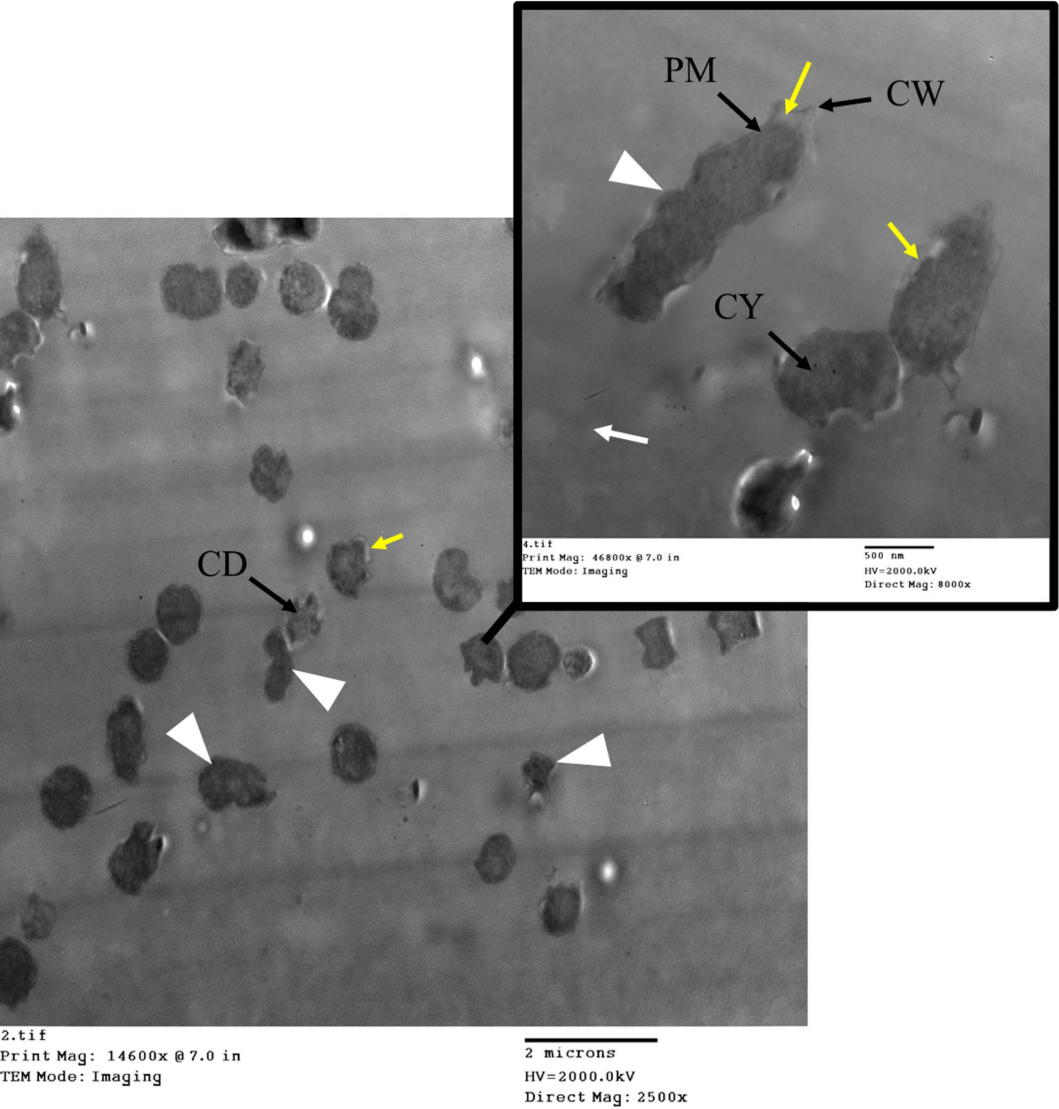
TEM imaging of *K. pneumoniae* after exposure to CDT. The images reveal significant ultrastructural disruption in the bacterial cells, which correlates with high cellular stress and potential cell death induced by the CDT environment. Yellow arrows point to cytoplasmic material leakage and dehydration. White arrowheads indicate highly irregular cells showing extreme pleomorphism. White arrows indicate membrane alterations, and areas of the cell membrane have ruptured. CD highlights regions showing abnormal cytoplasmic material, consistent with severe denaturation. CW is the cell wall. CM is the cytoplasmic membrane. CY is cytoplasm

Post-CDT TEM analysis of *K. pneumoniae* shows several structural changes (Fig. 15). The images revealed widespread morphological damage consistent with antibiotic-induced stress. The outer membrane appeared with slight damage with visible thinning, and the cytoplasm exhibited signs of stress or fragmentation. Observed features included numerous severely damaged and highly irregular cells, extreme cellular debris and evidence of lysis, and notable abnormality of the cytoplasmic material. These observational findings strongly correlate with the susceptibility/resistance phenotype of the isolate and illustrate the catastrophic impact of the test conditions on bacterial integrity.

## Discussion

It is known that patients in oncology wards have a higher risk of infection. These patients are more vulnerable to infection due to treatment and cancer. Infections are common among cancer patients and can delay treatment, increase medical costs, lengthen hospital stays, and lower survival rates. Despite a decline in mortality rates in recent years, infections remain a leading cause of death or closely linked causes. The majority of deaths caused by infections are caused by bacteria and fungi [13]. The management of infections is predicated on the application of appropriate empirical antimicrobial therapy and a comprehensive comprehension of commonly encountered microorganisms and antibiotic sensitivity patterns. As a consequence, the frequency of Gram-negative bacterial infections has decreased over the past two decades, while Gram-positive bacteria are now more frequently recognized. Even though the empirical use of antibiotics has reduced patient mortality, multidrug-resistant microorganisms continue to pose a threat. Immunocompromised individuals frequently encounter microorganisms that are resistant to many drugs [14]. Beta-lactamase-mediated bacterial resistance is a crucial mechanism for drug resistance in Enterobacteriaceae. Infections caused by Gram-negative bacteria, which produce beta-lactamases, can guide the selection of the most appropriate antibiotic [15]. This study aimed to identify three Gram-negative bacteria that were isolated from bladder cancer patients from various urine specimens.

The current study included antibiotic susceptibility testing after the bacterial isolates (*K. pneumoniae*, *E. coli*, and *P. aeruginosa*) were isolated and incubated. In this investigation, MEM was used to detect the three carbapenem-resistant Gram-negative bacilli (CRGNB) isolates (*K. pneumoniae*, *E. coli*, and *P. aeruginosa*) using the phenotypic MHT, mCIM, and CDT. Genotypic detection of several bacterial isolates utilizing the carbapenemase genes *blaNDM*, *blaVIM*, and *blaIMP*. Plasmid-mediated carbapenemase genes were examined in 35 *K. pneumoniae*, 10 *E. coli*, and 5 *P. aeruginosa* isolates out of 50. Lastly, agarose gel electrophoresis displays the outcomes of the polymerase chain reaction of the carbapenemase genes *blaNDM*, *blaVIM*, and *blaIMP*. The Gram-negative bacterial isolates included 35 *K. pneumoniae*, 10 *E. coli*, and 5 *P. aeruginosa* recovered from the different urine samples during the study. The resistance profile of *K. pneumoniae* and *E. coli* was greater than 80% for all tested antibiotics except AK, which showed reduced resistance, representing 80%. The resistance profile of *Pseudomonas* sp. exceeded 100% for all examined antibiotics. The results of this research indicate that AK is the preferred drug, as it was effective against *E. coli* and *K. pneumoniae*. Unfortunately, all other antibiotics that were examined exhibited a high level of resistance.

The TEM is a powerful imaging technique that provides high-resolution insights into the ultrastructural details of bacterial cells. It has been widely used in microbiological studies to examine the morphology, cellular architecture, and interactions of microorganisms. In the context of Gram-negative bacteria, TEM is particularly valuable for visualizing critical structures such as the cell envelope, pili, flagella, and membrane vesicles, which play pivotal roles in bacterial resistance mechanisms. This study employs TEM to investigate the structural adaptations and potential damage induced in *K. pneumoniae*, *E. coli*, and *P. aeruginosa* isolated from bladder cancer patients. By revealing ultrastructural changes associated with resistance, TEM contributes to a deeper understanding of how these pathogens evade antimicrobial treatments, offering insights into their pathogenicity and resistance. Cells exhibited outer membrane thinning, capsule disruption, or cytoplasmic disorganization. While these changes may reflect structural stress, mCIM itself does not necessarily expose cells to bactericidal meropenem concentrations capable of causing ultrastructural damage. Instead, the observed alterations may reflect intrinsic envelope fragility in carbapenem-resistant isolates (e.g., porin loss, efflux pump activity, capsule modifications). These interpretations align with Mourabiti et al. (2025), which reports that carbapenem interaction with penicillin-binding proteins can weaken cell walls and, in some cases, trigger autolysis under stress.

The majority of the isolates in this investigation were Gram-negative bacteria, in accordance with the microbiological results. These findings are in concordance with Ni et al. [16], who illustrated that they demonstrate a prevalence of this bacterial group and contrast with studies conducted in the 2000 s, which identified Gram-positive bacteria as the primary infectious agents. Alterations in epidemiology may result from the implementation of antibiotic prophylaxis, initiatives aimed at decreasing device-associated bacteremia, the increased intensity of chemotherapy regimens during the past decade, or the diminished utilization of chemotherapy catheters, among other factors. The present investigation demonstrated a substantial correlation between appropriate first antimicrobial therapy and survival rates. This outcome may stem from the judicious application of antimicrobials near the end of life, highlighting the necessity for interdisciplinary interventions to determine the length of antibiotic therapy in this patient cohort to mitigate their effects on the microbiota. This results in the same line as Cui et al. [17], who noted a correlation between insufficient antibiotic therapy and greater mortality rates, and with the fact that patients receiving palliative care also had a higher rate of death, our finding supports their claims.

The current study showed the resistance profile of *K. pneumoniae* and *E. coli was* greater than 80% for all tested antibiotics except AK, which showed reduced resistance, representing 80%. The resistance profile of *P. aeruginosa* exceeded 100% for all examined antibiotics. All other drugs that were tested showed significantly higher resistance rates. This study emphasizes that AK, which was effective against *E. coli* and *K. pneumoniae*, is the preferred medication. These study findings are similar to Langford et al. [18], who revealed that *A. baumannii*, *K. pneumoniae*, *E. coli*, and *P. aeruginosa* were the most often mentioned resistant Gram-negative bacteria. Antibiotic resistance was significantly higher in *A. baumannii* and *K. pneumoniae* compared to *E. coli* and *P. aeruginosa*. Furthermore, the colistin-resistant *K. pneumoniae* was found. *S. aureus* and *E. faecium* were the most often reported Gram-positive bacteria. Ampicillin, erythromycin, and CIP were all highly resistant against *E. faecium*.

In relation to the acquired resistance profile, the current study found that the isolates have acquired resistance. This result is in agreement with Selvaraj Anand et al. [19], who demonstrated a progressive increase in antimicrobial resistance; among the potential mechanisms, selective pressure resulting from improper antibiotic use has been identified. The current study demonstrates significant antibiotic resistance in bacteremia-inducing bacteria among cancer patients. Resistance rates were elevated in Gram-negative bacteria, particularly in *K. pneumoniae* isolates. The current investigation identified the key genes associated with resistant bacteria, notably *blaCTX-M* and *blaKPC*, which are prominent among extended-spectrum beta-lactamases and carbapenems, respectively. The anticipated consequence of resistance is a diminished likelihood of suitable empirical therapy, which influences the mortality and morbidity of these immunocompromised individuals. This result is contradictory to Vázquez-López et al. [20], who found that the results indicate that the bacteria exhibit a multidrug-resistant phenotype, especially when it comes to beta-lactam drugs. However, the main factor that determined this resistance was the presence of the *blaTEM* gene family. Out of the 209 positive strains (95.87%), 21.6% expressed it alone, and 21.6% expressed it in combination with other genes.

Concerning the reduced rate of *E. coli* isolation, the present research showed that it is still not obvious whether *E. coli* or another pathogen is the most common cause of UTIs in the community. This result mismatched with Rahman et al. [21], who reported that the *E. coli* isolate was the most prevalent in Gram-negative bacilli, with a 44.96% prevalence. Other significant isolates included *Enterobacter* (17.83%), *Klebsiella* sp. (14.72%), and *Citrobacter* sp. (12.4%). *Klebsiella* sp. was the second most prevalent organism in this investigation.

Regarding the prevalence of infection in relation to sex differences, the findings of the current investigation indicated that UTIs occur more in males than in females in bladder cancer patients. Nonetheless, various risk factors, such as age and sex, affect the infection prevalence differently. This result agrees with Chowdhury et al. [22]. A study demonstrated that after the age of 40, males were more susceptible to urinary tract infections (UTIs) among the elderly. There was a significant rise in the number of male patients aged 40–49. The risk of urinary tract infection likely increases as a result of the enlargement of the prostatic gland and the reduction of bacteriostatic prostatic secretions, which are related to aging. The findings contradict those of Forouzani et al. [23], who demonstrated that out of 702 urine samples (476 females and 226 males), 203 samples (28.92%) tested positive for urine culture and indicated urinary tract infections (UTIs). The cohort of patients with UTIs comprised 32.35% females (154 individuals) and 21.68% males (49 individuals). The prevalence rates of *E. coli*, *Klebsiella*, *Proteus*, and *Pseudomonas* were reported as 68%, 13%, 4%, and 2%, respectively.

Regarding MHT, mCIM, and CDT test results, the current study revealed that 82%, 32%, and 74% of isolated bacteria were carbapenem-resistant, respectively. PCR result R revealed that *blaIMP* is the most relevant gene, followed by *blaNDM* and finally *blaVIM*, with 36%, 32%, and 22%, respectively, as well as a positive result of PCR for *bla*NDM, *blaVIM*, and *blaIMP* individually. This result is consistent with Chelaru et al. [24], who reported that tigecycline and polymyxins are the only therapeutic options available to treat severe infections caused by NDM-1 producers, although the former may not achieve the necessary serum levels to treat systemic infections. In the same context, Herrera et al. [25]. mentioned that the MHT test was positive in 35% of Enterobacteriaceae isolates that were not susceptible to ertapenem. True-positive results were observed in 71% of these isolates, while 29% of them had inconclusive results. The IPM-EDTA-CDST phenotypic detection revealed that 75% of the isolates were MBL positive. The current study demonstrated that there are no significant variations in survival between individuals with an acquired resistance profile and those with natural susceptibility concerning the acquired resistance profile. This result is inconsistent with Riaño et al. [26], who identified a correlation between mortality and resistant bacteria. Additionally, age, comorbidities, and the inappropriate use of antimicrobials have been proposed as other factors related to mortality.

The phenotypic and molecular resistance patterns observed in cancer patients with bacteremia reveal significant geographic variability in bacterial resistance. Consequently, the findings of this study are particularly valuable, as they inform recommendations to enhance programs promoting the judicious use of antimicrobials and to advocate for the establishment or optimization of infection control committees in institutions treating cancer patients. This knowledge may assist in the development and deployment of molecular diagnostic tests in the country to discover bacterial resistance genes, serving as a valuable tool for informed antimicrobial decision-making. In the same context, Alemayehu et al. [27]. mentioned that individuals admitted to Soba University Hospital provided 206 GNB clinical specimens for analysis. Out of 206 carbapenem-resistant isolates, 107 (or 52%) had at least one carbapenemase gene, and 171 (or 83%) were found to be phenotypically resistant.

The current study showed the resistance profile of *K. pneumoniae* and *E. coli was* greater than 80% for all tested antibiotics except AK, which showed reduced resistance, representing 80%. The resistance profile of *P. aeruginosa* exceeded 100% for all examined antibiotics. This result disagreed with Kang et al. [28], who found that a total of 35 beneficial bacterial growths were identified, including *Stenotrophomonas maltophilia* and the notable *E. coli* (UPEC) strain. Of the microorganisms tested, 62.8% were found to be multidrug-resistant (MDR), while 28.5% were found to be XDR. In the same line, Bhat et al. [29]. found that of the organisms, 26.92% exhibit resistance. The percentage of *E. coli* and *K. pneumoniae* that produced ESBL was 50.4%. An 11.63% resistance rate to β-lactam and a 22.22% resistance rate to carbapenems were observed in Gram-negative organisms. Half of the *Staphylococcus* sp. strains were resistant to methicillin. A limitation of the present study is that our molecular analysis focused exclusively on the detection of metallo-β-lactamase genes. While these MBLs are of significant clinical concern, we did not conduct genetic testing for other important carbapenemase families. These include Class A or Class D oxacillinases, which are also highly prevalent. Although our phenotypic Combined Disk Test screened for Class A enzymes, the absence of broader genetic confirmation means the full molecular landscape of resistance in our isolates may not be completely captured. Future studies should incorporate a wider range. Furthermore, the study employed a convenience sampling strategy, which may introduce selection bias. Because isolates were not collected systematically (e.g., consecutively), our sample may not be fully representative of all Gram-negative isolates within the hospital. Therefore, the findings should be interpreted with this potential limitation in mind, and larger studies with systematic sampling are warranted to generalize our results. The study’s primary limitation is its small sample size, a result of recruitment challenges within a specific clinical population (bladder cancer patients) and its single-center nature. This limitation may reduce statistical power and generalizability, making our findings preliminary. We recommend future larger, multi-center studies to validate and expand upon these conclusions. A further limitation of this study is that the molecular detection of resistance genes relied solely on the results of conventional PCR. While this method is effective for screening for the presence of specific gene targets, we did not perform Sanger sequencing on the PCR amplicons to confirm their exact identity. The absence of sequence confirmation means that the possibility of non-specific amplification, while unlikely with optimized protocols, cannot be entirely ruled out. Therefore, future studies should incorporate sequencing to provide a more definitive characterization of the detected genes.

## Conclusions

In this study, the prevalence of carbapenemase-producing *K. pneumoniae*, *E. coli*, and *P. aeruginosa* isolates was a matter of great concern. Many of the antimicrobials employed in this study were highly resistant to isolates that produced carbapenemase. Carbapenemase-producing isolates were relatively susceptible to only AK. This study aimed to demonstrate the genotypic detection of *blaNDM*, *blaVIM*, and *blaIMP*. It was necessary to have a firm understanding of the presence of high-risk clones of antimicrobial resistance in carbapenemase genes that were present in Gram-negative isolates. TEM was implemented to investigate the ultrastructural morphology of specifically chosen multidrug-resistant isolates of *K. pneumoniae*,* E. coli*, and *P. aeruginosa*. TEM images revealed typical rod-shaped morphology with intact cell envelopes and well-defined cytoplasmic content. Notably, alterations in cell wall integrity and cytoplasmic density were observed in carbapenem-resistant isolates, which may be indicative of structural adaptations linked to resistance mechanisms. TEM was used to characterize antimicrobial-resistant Gram-negative pathogens and highlighted the value of ultrastructural analysis in understanding bacterial physiology under antibiotic pressure.

## Data Availability

All data generated or analyzed during this study were included in this article.

## Abbreviations

UTIs: Urinary tract infections
MHT: modified Hodge test
mCIM: Modified carbapenem inactivation method
CDT: Combined disk test
TEM: Transmission electron microscopy
GNB: Gram-negative bacteria
MIC: Minimum inhibition concentration
PCR: Polymerase chain reaction
TZP: Piperacillin-tazobactam
CTX: Cefotaxime
CAZ: Ceftazidime
FEP: Cefepime
IMP: Imipenem
MEM: Meropenem
CIP: Ciprofloxacin
AK: Amikacin
